# Efficacy and safety of four COVID-19 vaccines in preventing SARS-CoV-2 infection: A rapid review

**DOI:** 10.7705/biomedica.6254

**Published:** 2022-10-31

**Authors:** Lina Sofía Morón-Duarte, Kelly Rocío Chacón, María Paula Gutiérrez, Ilich Herbert De La Hoz, Nancy Yomayusa

**Affiliations:** 1 Instituto Global de Excelencia Clínica-Keralty, Grupo de Investigación Traslacional, Universidad Sanitas, Bogotá, D.C., Colombia Universidad Sanitas Bogotá D.C Colombia

**Keywords:** Coronavirus infections, vaccines, treatment outcome, safety, infecciones por coronavirus, vacunas, resultado del tratamiento, seguridad

## Abstract

**Introduction:**

Since the emergence of the SARS-CoV-2, there have been efforts to develop vaccines to control the COVID-19 pandemic.

**Objective:**

The present study assessed the efficacy and safety of the BNT162b2, mRNA-1273, ChAdOx1/AZD1222 and Gam-COVID-Vac rAd26-S/rAd5-S vaccines against the SARS-CoV-2.

**Materials and methods:**

We searched PubMed/MEDLINE, Google Scholar, Cochrane, and the WHO International Clinical Trials Registry Platform on March 15, 2021. The search terms used were: "vaccine" OR "vaccination" AND "covid19" OR "coronavirus" OR "sarscov2" AND "bnt162b2" OR "chadox1-S" OR "azd1222" OR "sputnik" OR "Gam-COVID-Vac" OR "mrna" OR "mRNA-1273" . We measured the risk of bias of the studies and the quality of the evidence using GRADE profiles. A qualitative and quantitative analysis of the results of clinical trials is presented.

**Results:**

Of the 74 identified studies, 4 were finally included in this review. The efficacies of the BNT162b2, mRNA-1273, ChAdOx1/AZD1222 and Gam-COVID-VacrAd26-S/rAd5-S vaccines against symptomatic COVID-19 were 95,0% (CI_95%_ 90,3-97,6), 94,1% (CI_95%_ 89,3-96,8), 66,7% (CI_95%_ 57,4-74,0), and 91,1% (CI_95%_ 83,8-95,1), respectively. There°was moderate certainty of the evidence due to serious indirectness, when we measured the risk of bias of the studies and the quality of the evidence using GRADE profile. The safety profiles were acceptable, and data on serious adverse events (summary RR=0,93; CI_95%_ 0,77-1,12; p=0,16) and deaths from all causes (summary RR=0,70; CI_95%_ 0,33-1,50; p=0°90) showed no significant differences.

**Conclusion:**

The results of this review support the level of evidence for the efficacy and safety of the COVID-19 vaccines analysed.

## Resumen

**Introducción.:**

Desde que surgió el virus SARS-CoV-2, se han realizado esfuerzos para desarrollar vacunas para controlar la pandemia por COVID-19.

**Objetivo.:**

Evaluar los datos de la eficacia y seguridad de las vacunas BNT162b2, mRNA-1273, ChAdOx1/AZD1222 y Gam-COVID-Vac rAd26-S/rAd5-S contra el SARS-CoV-2.

**Materiales y métodos.:**

Se realizaron búsquedas en PubMed/MEDLINE, Google Scholar, Cochrane y la Plataforma de Registro Internacional de Ensayos Clínicos de la OMS el 15 de marzo de 2021. Los términos usados fueron: "vaccine" OR "vaccination" AND "covid19" OR "coronavirus" OR "sarscov2" AND "bnt162b2" OR "chadox1-S" OR "azd1222" OR "sputnik" OR "Gam-COVID-Vac" OR "mrna" OR "mRNA-1273'.' Se midió el riesgo de sesgo de los estudios y la calidad de la información por medio de los perfiles GRADE. Se presenta un análisis cualitativo y cuantitativo de los resultados de los estudios clínicos.

**Resultados.:**

Se identificaron 74 estudios y se incluyeron 4 en la revisión. La eficacia de las vacunas BNT162b2, mRNA-1273, ChAdOx1/AZD1222 y Gam-COVID-VacrAd26-S/rAd5-S contra la COVID-19 sintomática fue del 95,0 % (IC_95%_ 90,3-97,6), 94,1 % (IC_95%_ 89,3-96,8), 66,7 % (IC_95%_ 57,4-74,0) y 91,1 % (IC_95%_ 83,8-95,1), respectivamente, y hubo una certeza moderada de la información debido a la falta de evidencia directa. Los perfiles de seguridad fueron aceptables, y los eventos adversos graves (RR resumido=0,93; IC_95%_ 0,77-1,12; p=0,16) y muerte por todas las causas (RR resumido=0,70; IC_95%_ 0,33-1,50; p=0,90) no mostraron diferencias significativas.

**Conclusión.:**

Los resultados de esta revisión respaldan el nivel de evidencia de la eficacia y seguridad de las vacunas COVID-19 que fueron analizadas.

The coronavirus disease 2019 (COVID-19) caused by the novel coronavirus 2019 (2019-nCoV) ^1^, also called severe acute respiratory syndrome coronavirus 2 (SARS-CoV-2) ^2^, has resulted in more than 160 million confirmed cases and 5 million deaths worldwide (November 29, 2021) ^3^. COVID-19 pandemic endangered global public health and economies and necessitated the development of effective vaccines to protect at-risk populations.

Containment measures failed to stop the spread of the virus, and there are no specific treatments against COVID-19. Vaccines prepare the immune system to detect and counteract viruses with greater success than other interventions. Successful cases of mass vaccination have controlled or eliminated smallpox, poliomyelitis, measles and rubella in America, which avoided illness, premature death and health costs ^4^.

Efforts to develop SARS-CoV-2 vaccines to control the pandemic have been underway since the virus emerged, with more than 500 candidate vaccines in clinical trials ^5^. Some vaccine development are more advanced than others. More than 80 candidate vaccines are being tested in humans ^6^. The impact of COVID-19 vaccines on the pandemic depends on several factors: the effectiveness of the vaccines; the speed of vaccine approval, manufacture, and delivery; the possible development of other variants; individual factors; and the number of people vaccinated ^7^.

The BNT162b2 (Pfizer BioNTech), mRNA-1273 (Moderna), ChAdOx1-S (Oxford University/AstraZeneca), and rAd26-S/rAd5-S (Gam-COVID-Vac) vaccines received emergency use authorization in some countries ^8^^-^^10^. The main countries where clinical trials of BNT162b2 vaccine were performed are the United States, Argentina, Brazil, Germany, South Africa, and Turkey. For ChAdOx1-S, the United Kingdom, South Africa and Brazil. The United States for mRNA-1273, and the Russian Federation for Gam-COVID-Vac rAd26-S/ rAd5-S. The World Health Organization (WHO) has also granted EUA to BNT162b2, mRNA-1273 and ChAdOx1-S vaccines ^11^.

Due to these public policy decisions on health, besides the promising preliminary results of these vaccines against the virus SARS-CoV-2 and the initiation of mass vaccination campaigns, it is necessary to evaluate the available evidence on its efficacy and safety.

## Materials and methods

We adhered to the Preferred Reporting Items for Systematic Reviews and Meta-Analyses (PRISMA) guidelines throughout the manuscript ^12^. The results were submitted to the International Register of Systematic Perspective Reviews (PROSPERO) and approved with registration number CRD42021229802. Two researchers performed the screening process independently and applied pre-established inclusion and exclusion criteria to select studies for a complete reading.

### Search strategy

On March15, 2021, we searched in PubMed/MEDLINE, Google Scholar, Cochrane, and the WHO International Clinical Trials Registry Platform. The search terms used were: "vaccine" OR "vaccination" AND "covid19" OR "coronavirus" OR "sarscov2" AND "bnt162b2" OR "chadox1-S" OR "azd1222" OR "sputnik" OR "Gam-COVID-Vac" OR "mrna" OR "mRNA-1273" These terms could be found anywhere in the article, title, or abstract. The search equations used for each of the databases are provided in online supplement 1. The Rayyan® web application was used to organise the list of references, remove duplicates, and obtain the full document for review.

#### Inclusion criteria

According to the PICOT question-model, population, intervention, comparison, and outcomes are shown in Table 1.

**Table 1:** Research questions using the PICOT framework

Populations	Persons with specific characteristics of each study
Interventions	Vaccines:
	BNT162b2 (Pfizer Biontech)
	mRNA-1273 (Moderna)
	ChAdOx1/AZD1222 (Oxford University/AstraZeneca)
	Gam-COVID-Vac rAd26-S/rAd5-S
Comparators	Placebo or any control arm, including no vaccine or other vaccines/alternate vaccine
Outcomes	Efficacy:
	• Symptomatic SARS-CoV-2 infection confirmed by laboratory
	• Hospitalization for COVID-19
	• Asymptomatic SARS-CoV-2 infection
	Safety:
	• Adverse events (any serious, moderate or mild adverse event)
	• Death from all causes

Studies: Randomized clinical trials (RCT) phases II/III and III in humans.

Results report: Studies that reported individual effect estimates for each primary research study that was attributable to the comparison of interest and at least one outcome.

- No language or country restrictions were applied.

### Exclusion criteria

We did not include studies that were only available on abstract format, because the information reported is insufficient to evaluate methodological quality.

### Data collection and analysis

#### Screening and selection of studies

Two reviewers screened the total number of references identified in the search by examining the titles and abstracts against independently predefined eligibility criteria. From the group of pre-selected references, a smaller number of studies were selected. The reviewers verified that each study met the eligibility criteria by reading the full text on each publication. Disagreements were resolved via consensus. To extract the information, a standardized Excel tool was used, which was tested by the reviewers before use. The structure was based on collecting information on the basic characteristics of each study, such as participants, intervention (vaccine), comparators and outcomes. The data extraction was performed in duplicate and subsequently verified by the researchers involved, who compared the extracted data with the studies.

Reviewers selected the effect estimates of for the comparison and critical outcomes from reported values in the studies.

#### Assessments of risk of bias and certainty of the evidence

The Cochrane risk of bias tool was used to assess the following domains: randomization, deviations from intended intervention, missing outcome data, measurement of the outcome, selection of the reported results, and overall risk of bias ^13^. Studies were categorized as: "high risk", "low risk" or "some concerns" The certainty of the evidence was measured using GRADE profiles ^14^. A single-first reviewer author rated the risk of bias and certainty of the evidence for each study, and a second reviewer author checked the ratings.

### Statistical analysis

Abstracted data were aggregated in tables. Risk ratios (RR), risk differences and corresponding 95% confidence intervals (CI_95%_) were calculated or extracted from the selected publications. We computed the RR for the outcomes of the adverse events and serious adverse events. Data on the vaccine efficacy were extracted from the publications and calculated ([1-(risk ratio or rate ratio comparing vaccine and placebo recipients)] x 100) for the outcome asymptomatic or unknown infection of the vaccine ChAdOx1-S.

We performed inverse variance-weighted random-effects meta-analyses using the Paule and Mandel **t** 2 estimator for heterogeneity ^15^. Heterogeneity across the RCTs was described using the I 2 and **t** 2 metrics ^16^. Data were analysed using the statistical package Stata™, version 15 (Stata Corporation, College Station, Texas, United States).

## Results

Figure 1 shows the search results, screening, and selection of evidence for this rapid review. The search of the identified databases detected 74 documents. After exclusion of duplicates, 68 articles remained for title and abstract screening. Fifty-six of these papers were deemed irrelevant according to the titles and abstracts and were later excluded. We assessed the full text of the remaining 12 articles: seven of these articles were excluded due to RCT phase I (n=1), phase I/II (n=4), phase II/III (n=1, no outcomes of interest), and wrong vaccine and phase I/II (n=1). Bibliographic data of these 7 studies were excluded after full-text assessment 17-23. Of the 5 included articles, two reports were from the same trial (ChAdOx1-S vaccine). When outcomes were reported in both publications, the updated version was used. A total of 4 RCTs were included, and all studies were performed in high- or middle-income countries, such as the United States, Argentina, Brazil, Germany, South Africa, Turkey, United Kingdom and the Russian Federation. There were 105,118 participants (57,591 randomized to the vaccine against COVID-19 and 47,527 to placebo) 24-28. The age of the participants at study entry ranged from 16 to 85 years. We summarized the characteristics of the studies included in Table 2.

**Figure 1:**
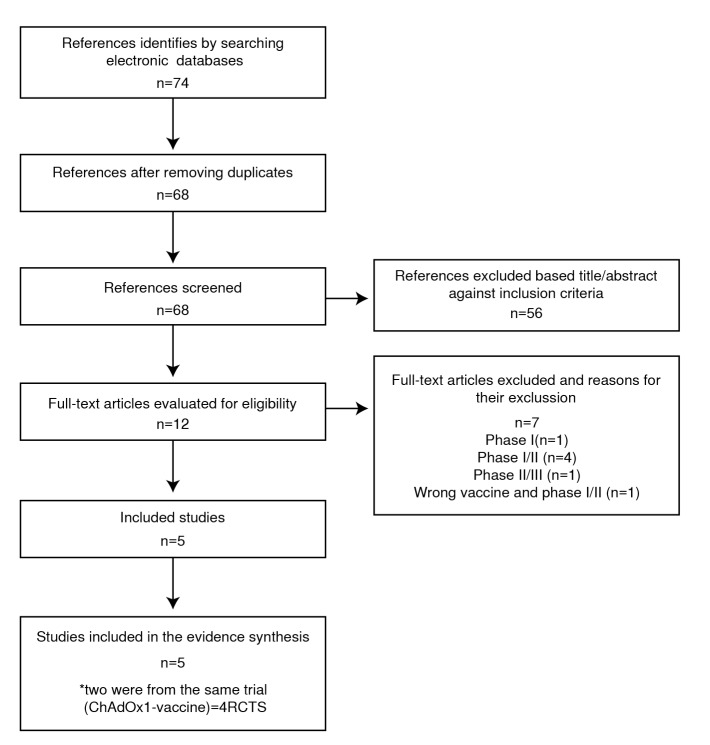
Flowchart of study selection conforming to PRISMA 12

**Table 2:** Characteristics of the included studies

Study	Polack, 2020 [24]	Baden, 2020 [25]	Voysey, 2020 [26] [27]	Logunov, 2021 [28]
Vaccine platform description	RNA based vaccine	RNA based vaccine	Wnr, Viral vector (Non-replicating)	Wnr, Viral vector (Non-replicating)
Phase	III	III	III	III
Population/sample size in phase III clinical trial recruitment	Persons aged ≥16 years/37.724	Persons aged 18≥years/30.351	Persons aged 18≥years/17.177	Persons aged 18≥years/19.866
Intervention	Pfizer-BioNTech COVID-19 vaccine BNT162b2 (30 μg, 2 doses IM, 21 days apart)	Moderna COVID-19 vaccine mRNA -1273 (100 μg, 2 doses IM, 28 days apart)	AstraZeneca + University of Oxford COVID-19 vaccine ChAdOx1-S - (AZD1222) (Covishield) (2 doses IM, 28 days apart)	Gam-COVID-Vac combined vector vaccine, 0.5 ml/ dose+0.5 ml/dose prime-boost immunization in days 1 (component I rAd26-S) and 21(component II rAd5-S)
Comparison	No Pfizer-BioNTech COVID-19 vaccine/placebo	No COVID-19 vaccine	Meningococcal Group A, C, W, and Y conjugate vaccine or (MenACWY) saline	Placebo, 0.5 ml/dose+0.5 ml/dose immunization in days 1 and 21
Countries	United States; Argentina; Brazil; Germany; South Africa; Turkey	United States	United Kingdom, South Africa, and Brazil	Russian Federation
No. of patients by groups				14,964
Vaccine group	18,860	15,170	8,597	
Placebo group	18,864	15,181	8,580	4,902
Age median or **mean (SD)**				
Vaccine group	52 (16-89)	51.4 (18-95)	NR	45.3 (SD=12.0)
Placebo group	52 (16-91)	51.3 (18-95)	NR	45.3 (SD=11.9)
Percentage of the population at high risk (>55 years) **Sex No. (%)**	40.90%	42%	12.20%	34.2% (>51 years)
Vaccine group				
Male	9,639 (51.1)	7,923 (52.2)	3,779 (44.0)	9,143 (61.1)
Female	9,221 (48.9)	7,258 (47.8)	4,816 (56.0)	5,821 (38.9)
Placebo group				
Male	9,436 (50.1)	8,062 (53.1)	3,601 (42.0)	3,015 (61.5)
Female	9,410 (49.9)	7,108 (46.9)	4,980 (58.0)	1,887 (38.5)
Comorbidities				
Vaccine group	with any Charlson comorbidity: 3,934 (20.9)	Chronic lung disease: 710 (4.7); severe obesity: 1.025 (6.8); diabetes:1,435 (9.5); liver disease: 100 (0.7)	Cardiovascular disease: 1,040/8,241 (12.6); respiratory disease: 872/8,241 (10.6); diabetes: 237/8,241(2.9)	Concomitant diseases (diabetes, hypertension, ischaemic heart disease, obesity): 3,687/14,944 (24.7)
Placebo group	with any Charlson comorbidity: 3,809 (20.2)	Chronic lung disease: 744 (4.9); severe obesity: 1,021 (6.7); diabetes: 1,440 (9.5); liver disease: 96 (0.6)	Cardiovascular disease: 999/8,196 (12.2); respiratory disease: 872/8,196 (10.6); diabetes: 23/8,196 (2.5)	Concomitant diseases (diabetes, hypertension, ischaemic heart disease, besity): 1,235/4.892 (25.2)

SD: Standard deviation

### Risk of bias

The overall risk of bias was classified as having some concerns in all 4 RCTs due to deviations in the interventions. In general, all trials assessed the main efficacy outcomes using per-protocol and not intention-to-treat analyses. Details of the risk of bias assessment are provided in online supplement 2.

### Data availability

Symptomatic SARS-CoV-2 infection confirmed by laboratory, severe or critical disease due to COVID-19, serious adverse events and all-cause mortality were assessed in all 4 RCTs ^24^^-^^28^ (Tables 3 and 4). The outcomes of hospitalization for COVID-19 and asymptomatic SARS-CoV-2 infection were reported in one RCT (Tables 3 and 4) ^26^^,^^27^.

**Table 3:** Efficacy of vaccination against SARS-CoV-2 infection (COVID-19)

Symptomatic SARS-CoV-2 infection confirmed by laboratory													Severe disease due to COVID-19			Asymptomatic or unknown infection			Hospitalization due to COVID-19		
Study	n of events/n of participants		VE (CI_95%_)	n of events/n of participants		VE (CI_95%_)	n of events/n of participants		VE (CI_95%_)	n of events/n of participants		VE (CI_95%_)	n of events/n of participants		VE (CI_95%_)	n of events/n of participants		VE (CI_95%_)	n of events/n of participants		VE (CI_95%_)
	Vaccine	Placebo	After dose 2	Vaccine	Placebo	After dose 1	Vaccine	Placebo	From 14 days after dose 1	Vaccine	Placebo	From day 22 to day 90 after dose 1	Vaccine	Placebo		Vaccine	Placebo		Vaccine	Placebo	
Polack, 2020 [24]	8/17,411	162/17,511	95.0% (90.3 to 97.6)*	50/21,669	275/21,686	82.0% (75.6 to 86.9)	2/21,669	27/21,686	92.6% (69.0 to 98.3)	NA	NA	NA	1/21,314	9/21,259	88.9% (12.5 to 98.6)	NA	NA	NA	NA	NA	NA
Baden, 2020 [25]	11/14,134	185/14,073	94.1% (89.3 to 96.8)**	NA	NA	NA	2/14,550	35/14,598	94.3% (76.2 to 98.6)	NA	NA	NA	0/14,134	30/14,073	100%	NA	NA	NA	NA	NA	NA
Voysey, 2021 [26] [27]	84/8,597	248/8,580	66·7% (57.4 to 74.0)**	NA	NA	NA	NA	NA	NA	17/9,257	71/9,237	76.0% (59.3- 85.9)	0/12,021	1/11,724	100%	41/2,692	42/2,751	2·0% *** (−50·7 to 36·2)	0/11,794	15/11,776	100%
																16/1,379	31/1385,	49·3%**** (7·4 to 72·2) 22.2% (-9.9 to 45.0)****			
																57/4,071	73/4,136				
Logunov, 2021 [28]	13/14,094	47/4,601	91.1% (83.8 to 95.1)*	16/14,964	62/4,902	91.6% (85.6 to 95.2)	30/14,999	79/4,950	87·6% (81.1 to 91.8)	NA	NA	NA	0/14,964	20/4,902	100%	NA	NA	NA	NA	NA	NA

*7 days after the second dose; ** 14 days after the second dose; VE: vaccine efficacy; NA: not applicable; ***Two standard doses; ****Low dose plus standard dose; ***** All doses

**Table 4:** Vaccine efficacy according to age, sex, and race subgroup

Age group	Polack, 2020 [24]			Age group	Baden, 2020 [25]			Voysey, 2021[26] [27]				Logunov, 2021 [28]		
	BNT162b2 (n=18,198)	Placebo (n=18,325)	VE (95% CI)		mRNA-1273 (n=14,134)	Placebo (n=14,073)	VE (CI_95%_)	ChAdOx1	Placebo	VE (CI_95%_)	Age group	Gam-COVID-Vac rAd26-S/rAd5-S (N=14,964)	Placebo (N=4,902)	VE (CI_95%_)
16 to 55 yr	5/9,897	114/9,955	95.6 (89.4 to 98.6)	≥18 - <65 yr	7/10,551	156/10,521	95.6 (90.6 to 97.9)	NR	NR	NR	18-30	1/1,596	4/521	91.9% (51.2 to 99.3)
>55 yr	3/7,500	48/7,543	93.7 (80.6 to 98.8)	≥65 yr	4/3,583	29/3,552		NR	NR	NR	31-40	4/3,848	13/1,259	90.0% (71.1 to 96.5)
≥65 yr	1/3,848	19/3,880	94.7 (66.7 to 99.9)				86.4 (61.4 to 95.2)	NR	NR	NR	41-50	4/4,399	15/1,443	91.3% (73.7 to 96.9)
≥75 yr	0/774	5/785	100.0 (−13.1 to 100.0)					NR	NR	NR	51-60	5/3,510	22/1,146	92.7% (81.1 to 97.0)
											>60	2/1,611	8/533	91.8% (67.1 to 98.3)
Sex				Sex							Sex			
Male	3/8,875	81/8,762	96.4 (88.9 to 99.3)	Male	4/7,366	87/7,462	95.4(87.4 to 98.3)	NR	NR	NR	Male	7/9,143	39/3,015	94.2% (87.2 to 97.4)
Female	5/8,536	81/8,749	93.7 (84.7 to 98.0)					NR	NR	NR	Female	9/5,821	23/1,887	87.5% (73.4 to 94.2)
				Female	7/6,768	98/6,611	93.1 (85.2 to 96.8)	NR	NR	NR				
Race or ethnic group				Race or ethnic group										
White	7/14,504	146/14,670	95.2 (89.8 to 98.1)	White	10/9,023	144/8,916	93.2 (87.1 to 96.4)	NR	NR	NR	NR	NR	NR	NR
Black or African American	0/1,502	7/1,486	100.0 (31.2 to 100.0)					NR	NR	NR	NR	NR	NR	NR
Hispanic or Latin	–,764	53/4,746	94.4 (82.7 to 98.9)	Communities of colour	1/5,088	41/5,132	97.5 (82.2 to 99.7)	NR	NR	NR	NR	NR	NR	NR
Non-Hispanic, non-Latin	5/12,548	109/12,661	95.4 (88.9 to 98.5)					NR	NR	NR	NR	NR	NR	NR
All others	1/1,405	9/1,355	89.3 (22.6 to 99.8)					NR	NR	NR	NR	NR	NR	NR

VE: vaccine efficacy; NR: Not reported; Yr: years old

### Efficacy outcomes

The efficacies of the vaccines BNT162b2, mRNA-1273, ChAdOx1-S and Gam-COVID-VacrAd26-S/rAd5-S against SARS-CoV-2 infection (COVID-19) via assessment of symptomatic SARS-CoV-2 infection confirmed by a laboratory after the second dose were 95.0% (CI_95%_ 90.3-to 97.6), 94.1% (CI_95%_ 89.3-96.8), 66.7% (CI_95%_ 57.4-74.0) and 91.1% (CI_95_%, 83.8-95.1), respectively ° (24-28). The efficacy results after the first doses were also reported (Table 3). The efficacy of vaccine BNT162b2 against severe disease due to COVID-19 was 88.9% (CI_95%_, 12.5 to 98.6), and 100% for the other vaccines (mRNA-1273, ChAdOx1-S and Gam-COVID-VacrAd26-S/rAd5-S) ^24^^-^^28^. The efficacy of the vaccine ChAdOx1 in asymptomatic or unknown SARS-CoV-2 infection participants was 22.2% (CI_95%_ -9.9 to 45.0), and 100% in hospitalized patients with COVID-19 (Table 3) ^26^^,^^27^. Vaccine efficacy in subgroups defined by age, sex, and race was generally consistent with the overall population (Table 4). Efficacy meta-analysis was not performed because RCTs assessed this outcome differently.

### Adverse events

More vaccine recipients than placebo recipients reported any serious moderate or mild adverse events (Table 5) ^24^^-^^28^. Serious adverse events did not show statistically significant differences between the analysis groups. Across the 4 RCTs, the summary RR for serious adverse events was 0.93 (CI_95%_ 0.77-1.12; p=0.16). The heterogeneity between trials was (I2=42.5%; **t**2=0.02; p=0.424). Reports of deaths from all causes were rare. Across the 4 RCTs, the summary RR for all-cause mortality with vaccines against SARS-CoV-2 infection was 0.70 (CI_95%_ 0.33-1.50; p=0.90). There was no significant difference between-trial heterogeneity (I2=0%; **t**2=0; p=0.358) (Figures 1 s and 2 s in online Supplement 3). No participant who received any vaccine died of COVID-19 ^24^^-^^28^. There was one death from COVID-19 in the placebo group in the RCT of the mRNA-1273 vaccine ^25^.

**Table:** 5. Number of participants reporting any event and serious adverse events

Adverse event	Polack, 2020 [24]		Baden, 2020 [25]		Voysey, 2021 [26] [27]		Logunov, 2021 [28]	
	BNT162b2 (n=21,621)	Placebo (n=21,631)	mRNA-1273 (n=15,185)	Placebo (n=15,166)	ChAdOx1 (n=12,282)	Placebo (n=11,962)	Gam-COVID-Vac rAd26-S/ rAd5-S (n=16,427)	Placebo (n=5,435)
Number of participants reporting any event	5,770 (26.7%)	2,638 (12.2%)	3,632 (23.9%)	3,277 (21.6%)	95/12,021 (0.8%)	126/11,724 (1.1%)		
	RR=2.19		RR=1.11		RR=0.74			
	(2.10 to 2.28)		(1.06 to 1.15)		(0.56 to 0.96)			
Related	4,484 (20.7%)	1,095 (5.1%)						
Sever**e**	240 (1.1%)	139 (0.6%)						
Number of participants reporting any serious adverse events	126 (0.6%)	111 (0.5%)	153 (1.0%)	147 (1.0%)	108 (0.9%)	127 (1.1%)	45 (0·3%)	23 (0·4%)
Related	4 (0.0%)	0 (0.0%)						
Severe	71 (0.3)	68 (0.3)	234 (1.5)	202 (1.3)			0 (0.0%)	
	2/21,621	4/21,631	6/15,185	7/15,166	1/12.282	4/11,962	3/16,427	1/5,435
All-cause mortality	RR=0.50		RR=0.86		RR=0.49		RR=0.99	
	(0.09 to 2.73)		(0.29 to 2.55)		(0.09 to 2.66)		(0.10 to 9.54)	

RR: Risk ratios

### Certainty of the evidence

For the efficacy outcome evaluated via symptomatic SARS-CoV-2 infection confirmed by a laboratory, the certainty of the evidence (using GRADE) was moderate due to serious indirectness (data from an interim analysis of the trial, with a short follow-up duration; estimates may change during longer follow-up; population included in RCTs may not represent all persons aged 16 years in BNT162b2; and 18 years in mRNA-1273, ChAdOx1-S, Gam-COVID-VacrAd26-S/rAd5-S). For the severe or critical disease due to COVID-19 in the RCTs regarding BNT162b2 and ChAdOx1 vaccines, the certainty of the evidence was very low, and moderate for mRNA-1273 and Gam-COVID-VacrAd26-S/rAd5-S vaccines. In the four RCTs, the certainty of the evidence for the outcomes of all-cause mortality and serious adverse events was very low due to serious indirectness and very serious imprecision based on the wide CI_95%_ for RR, which is consistent with substantial benefit or harm (see GRADE tables for more details in online supplement 4).

## Discussion

Available data indicated that the vaccines evaluated in this systematic review effectively prevented symptomatic laboratory-confirmed COVID-19 (range 69.7-95%) ^24^^-^^28^, with moderate certainty of evidence. For severe cases of the disease, the four evaluated vaccines were between 90and 100% effective with moderate to very low certainty of evidence. The subgroup analysis in each RCT revealed that the global efficacy of the vaccine was similar to the efficacy showed in different populations according to age, sex, comorbidities, and race/ethnicity ^24^^-^^28^.

The current concern related to asymptomatic infections is based on people who is able to continue transmitting the virus to others despite being vaccinated. Although asymptomatic infections are not a direct measure of disease transmission, researchers have viewed the information on this result as an indicator of how vaccines would help reducing the risk of the spread of SARS-CoV-2. The reports included in this review revealed information on this outcome from the ChAdOx1-S vaccine, which provided preliminary data on the prevention of asymptomatic SARS-CoV-2 infection. Showing an efficacy of 49.3% (CI_95%_ 7.4-72.2) in the group that received a half dose followed by a standard dose, compared to only 2% (CI_95%_ -50.7-36.2) in the group that received two standard doses ^26^^,^^27^. These results suggest that COVID-19 vaccines also reduce asymptomatic infection and potentially transmission.

The evidence shows that vaccination prevents a person from getting COVID-19 in its severe form and reduces the possibility of transmission to others ^29^. Substantial reductions in SARS-CoV-2 infections (symptomatic and asymptomatic) will help to reduce overall levels of disease and the transmission of the virus worldwide.

For adverse events and death, the analysed vaccines showed an adequate safety profile. However, the level of certainty of evidence was very low. Therefore, surveillance and follow-up of vaccinated cohorts is very important for the identification of possible adverse effects, especially rare events of death or disability.

Our review synthesized the evidence in a simple and methodical way to inform health providers and readers about the general efficacy and safety of four vaccines against COVID-19. These vaccines exhibited different degrees of effectiveness in the prevention of SARS-CoV-2 infection. However, all were very effective in preventing serious forms of illness and secondary death from COVID-19. The BNT162b2, mRNA-1273, ChAdOx1-S, and Gam-COVID-VacrAd26-S/rAd5-S vaccines showed high efficacy based on the minimum criteria established by the WHO to recommend a vaccine against COVID-19, which consisted of an estimate of 50% and with a clear demonstration of efficacy in the base population ^30^.

Global, national and regional regulatory procedures evaluating the suitability of new medical devices for public health emergencies, are responsible for performing a rigorous process to decide the administration of a vaccine with specified prioritisation for the earliest use ^31^. Other vaccines that were not the subject of this review have also received approval for emergency use by health regulatory agencies in different countries, such as CanSino, Sinopharm, Sinovac and Johnson & Johnson's vaccines ^32^^-^^35^. This information is encouraging due to the main challenge producing safe and effective vaccines in sufficient quantity for equitable distribution worldwide. More vaccines provide greater hope of ending the pandemic.

### Strengths and limitations

Rapid review is a methodology that may be particularly important in the COVID-19 pandemic because the evidence is rapidly emerging and verified information is needed to make policy or practical decisions. However, there are some limitations to our study, which primarily relate to the performance of this review in a limited time frame. First, while a comprehensive search was performed in three databases, there was insufficient time to search other sources (grey literature) to ensure comprehensiveness. However, making these methodological trade-offs is consistent with the time-limited approach taken in other rapid review methodologies ^36^^,^^37^.

Second, this review included only 4 phase II/III and III clinical trials with preliminary published data because they involve ongoing research. We did not include other study designs that analysed real-life data, which provide more reliable and representative conclusions for the population. Third, there is no evidence of the long-term effectiveness and safety of the vaccine.

These trials had a short follow-up of up to 28 days after vaccination and unsolicited serious adverse events through 6 months after the second dose. It is necessary to highlight that the level of evidence of the outcomes evaluated in this review ranged between moderate and very low. This is primarily because the analysis was performed per-protocol, as planned for the interim analysis, which generates uncertainty in the results because the estimates may change during longer follow-up. The results of an RCT acquire greater validity when the patients are analysed according to the group in which they were assigned, i.e., application of the intention-to-treat principle. This application makes it possible to maintain the advantages of randomization, avoiding overestimates of the effects of the therapy under study, and admitting the non-adherence of some patients, which is a situation that is closer to reality. Finally, we found no major limitations in study designs in relation to randomization sequence, blinding of investigators, personnel involved in the study, or significant losses to follow-up. However, the sample sizes of the intervention group (n=14,964) and the control group (n=4,902) (Table 3) in the RCT of the Gam-COVID-VacrAd26-S/rAd5-S vaccine were striking, but the distributions observed of the baseline characteristics between the study groups ^28^ were compatible with chance.

### Implications for practice

Health care providers and professionals should communicate consistent, complete, and clear information on the benefits, risks, contraindications, safety issues, warnings and follow-up recommendations to persons receiving COVID-19 vaccines and their caregivers. It should be explained that there is limited evidence on how much COVID-19 vaccines reduce transmission ingeneral population and for how long protection lasts. Also, prevention and biosecurity guidelines should be strictly followed. This includes the use of masks, hand washing, social distance greater than 2 meters, avoiding crowdsand enclosed unventilated areas ^38^^,^^39^.

We should generate pharmacovigilance systems to identify and respond quickly to any adverse events in a recipient after vaccination, including vaccine administration errors, serious adverse events, multisystemic inflammatory syndrome cases, and COVID-19 cases resulting in hospitalization and death. It is recommended that any other clinically significant adverse event should be reported, even if its association with the administration of the vaccine is not clear.

### Implications in research

It is necessary to keep track of the studies and reports that evaluate the beneficial and harmful effects of the different vaccines, which may be possible using living systematic reviews periodically informing the best practices in vaccine prevention and clinical research of this highly prevalent disease.

The results of this review support the level of evidence for the efficacy and safety of the COVID-19 vaccines that were analysed. The information presented in this manuscript has not been presented elsewhere.

## Supplementary material

**Supplement 1:** Evidence search reports in electronic databases

Data source	Search equation
PubMed/MEDLINE	((((("vaccine"[All Fields] OR "vaccination"[All Fields]) AND "covid19"[All Fields]) OR "coronavirus"[All Fields] OR "sarscov2"[All Fields]) AND "bnt162b2"[All Fields]) OR "chadox1"[All Fields] OR "azd1222"[All Fields] OR "sputnik"[All Fields] OR "mrna"[All Fields]) AND ((randomizedcontrolled trial[Filter]) AND (2020:2021[pdat]))
Cochrane	#1 Covid19
Embase	#2 vaccines in Trials #3 #1 AND #2 #4 bnt162b2 mrna vaccine #5 chadox1 #6 azd1222 #7 Gam-COVID-Vac #8 sputnik3 #9 mRNA-1273 #10 #4 OR #5 OR #6 OR #7 OR #8 OR #9 #11 #3 AND # 10 #12 #1 AND #2 AND # 10
Google Scholar	“vaccine" OR "vaccination" AND “Covid19”

**Supplement 2:** Risk of bias in randomized controlled trials

Study	Randomisation	Deviations from intervention	Missing outcome data	Measurement of the outcome	Selection of the reported results	Overall risk of bias	Outcome	Details
Polack, 2020 ^24^ BNT162b2	+	±	+	+	+	±	Low risk of bias: Adverse events; death from all causes Some concerns risk: Symptomatic SARS-CoV-2 infection confirmed by laboratory	Analysed by perprotocol and not by intention to treat; losses to follow-up were not described.
Baden, 2020 ^25^ mRNA-1273	+	±	+	+	+	±	Low risk of bias: Adverse events; death from all causes Some concerns risk: Symptomatic SARS-CoV-2 infection confirmed by laboratory and severe case of COVID-19	Analysed by perprotocol and not by intention to treat
Voysey, 2020 ^26^ ChAdOx1	+	±	+	+	+	±	Low risk of bias: Adverse events; death from all causes Some concerns risk: Symptomatic SARS-CoV-2 infection confirmed by laboratory	Analysed by per- protocol and not by intention to treat; Vaccine administration errors
Logunov, 2021 ^28^^)^ Gam-COVIDVac rAd26-S/ rAd5-S	+	±	+	+	+	±	Some concerns: Symptomatic SARS-CoV-2 infection confirmed by laboratory; severe case of COVID-19; Adverse events; death from all causes	Analysed by per- protocol and not by intention to treat; Vaccine administration errors

## Supplement 3

**Figure 1s:**
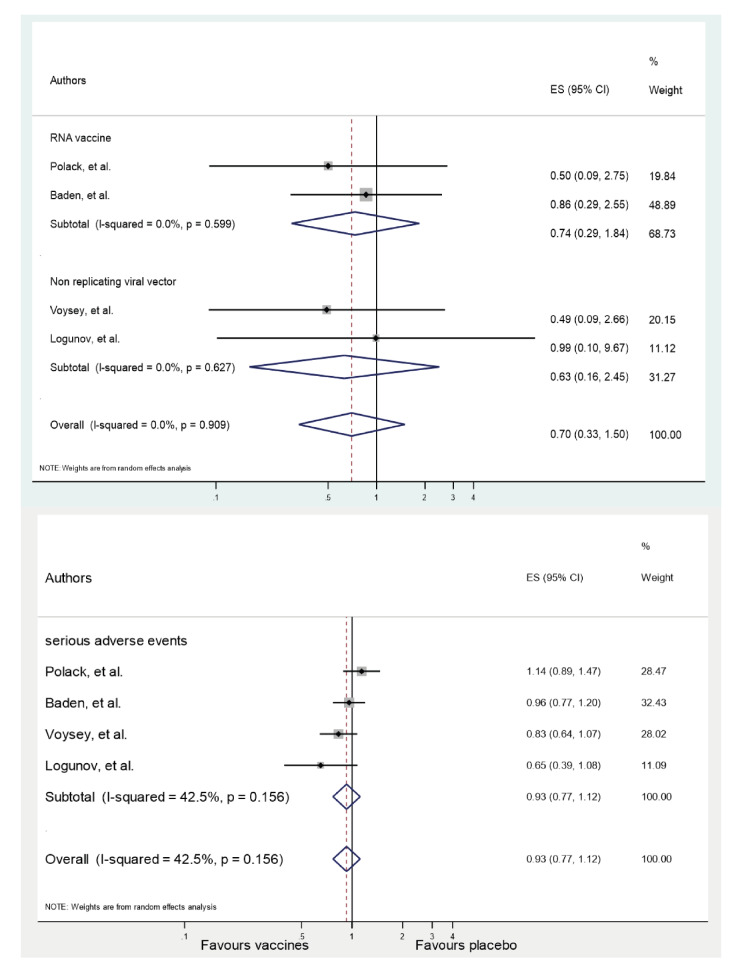
Forest plots with summary effect sizes per vaccine platform type: all-cause mortality

**Supplement 4s:** GRADE assessment

	Certainty assessment							No. of patients		Effect		Certainty	Importance
	No. of studies	Design	Risk of bias	Inconsistency	Indirectness	Imprecision	Other considerations	Vaccine	Placebo	Relative (95% CI)	Absolute (95% CI)		
Polack, 2020 ^15^ Vaccine: BNT162b2	1	Randomized trials	Not serious	Not serious	Serious^a b^	Not serious	None	8/17,411	162/17,511	RR=0.05 (0.02 to 0.10)		⨁⨁⨁◯ Moderate	Critical
Baden, 2020 ^16^^)^ Vaccine: mRNA-1273	1	Randomized trials	Not serious	Not serious	Serious^a b^	Not serious	None	11/14,134	185/14,073	RR=0.06		⨁⨁⨁◯ Moderate	Critical
Voysey, 2020 ^17^^,^^18^ Vaccine: ChAdOx1	1	Randomized trials	Not serious	Not serious	Serious^a b^	Not serious	None	84/8,597	248/8,580	RR=0.33 (0.26 to 0.43)		⨁⨁⨁◯ Moderate	Critical
Logunov, 2021 ^19^ Vaccine: Gam-COVIDVac rAd26-S/ rAd5-S	1	Randomized trials	Not serious	Not serious	Serious^a b^	Not serious	None	13/14,094	47/4,601	RR=0.09 (0.05 to 0.16)		⨁⨁⨁◯ Moderate	Critical

CI: Confidence interval; RR: Risk ratio Explanations

a. Data are from an interim analysis of the trial, with a short duration of follow-up. Estimates may change over a longer duration of follow-up.

b. The population included in the RCT may not represent all persons aged ≥16 years (BNT162b2) or ≥18 years (mRNA-1273; ChAdOxl; Gam-COVID-Vac rAd26-S/rAd5-S)

### Severe or critical disease due to COVID-19

**Table:** 

	Certainty assessment							No. of patients		Effect		Certainty	Importance
	No. of studies	Design	Risk of bias	I**nconsistency**	Indirectness	Imprecision	Other considerations	Vaccine	Placebo	Relative (95% CI)	Absolute (95% CI)		
Polack, 2020 ^24^ Vaccine: BNT162b2	1	Randomized trials	Not serious	Not serious	Serious^a b^	Very serious ^c^	None	1/21,314	9/21,259	RR=0.11 (0.01 to 1.81)		⨁◯◯◯ Very low	Critical
Baden, 2020 ^25^ Vaccine: mRNA-1273	1	Randomized trials	Not serious	Not serious	Serious^a b^	Not serious	None	0/14,134	30/14,073	RR=0.02 (0.00 to 0.27)		⨁⨁⨁◯ Moderate	Critical
Voysey, 2020 ^26^^,^^27^ Vaccine: ChAdOx1	1	Randomized trials	Not serious	Not serious	Serious^a b^	Very serious ^c^	None	0/12,021	1/11,724	RR=0.33 (0.01 to 7.98)		⨁◯◯◯ Very low	Critical
Logunov, 2021 ^28^ Vaccine: Gam-COVID- Vac rAd26-S/ rAd5-S	1	Randomized trials	Not serious	Not serious	Serious^a b^	Very serious ^c^	None	0/14,964	20/4,902	RR=0.00 (0.00 to 0.06)		⨁◯◯◯ Very low	Critical

CI: Confidence interval; RR: Risk ratio Explanations

Data are from an interim analysis of the trial, with a short duration of follow-up. Estimates may change over a longer duration of follow-up.

The population included in the RCT may not represent all persons aged ≥16 years (BNT162b2) or ≥18 years (mRNA-1273; ChAdOxl; Gam-COVID-Vac rAd26-S/rAd5-S) Imprecision was downgraded by 2 levels because the 95% of the relative risk (RR) was sufficiently wide that the estimate could include appreciable harm or benefit of the intervention. This outcome may be imprecise due to the small number of events reported during the observation period.

### Any adverse event

**Table:** 

	Certainty assessment							No. of patients		Effect		Certainty	Importance
	No. of studies	Design	Risk of bias	I**nconsistency**	Indirectness	Imprecision	Other considerations	Vaccine	Placebo	Relative (95% CI)	Absolute (95% CI)		
Polack, 2020 ^15^ Vaccine: BNT162b2	1	Randomized trials	Not serious	Not serious	Serious^a b^	Not serious	None	5,770/21,621	2,638/21,631	RR=2.19 (2.10 to 2.28)		⨁⨁⨁◯ Moderate	Critical
Baden, 2020 ^16^ Vaccine: mRNA-1273	1	Randomized trials	Not serious	Not serious	Serious^a b^	Not serious	None	3,632/15,185	3,277/15,166	RR=1.11 (1.06 to 1.15)		⨁⨁⨁◯ Moderate	Critical
Voysey, 2020 ^17^^,^^18^ Vaccine: ChAdOx1	1	Randomized trials	Not serious	Not serious	Serious^a b^	Not serious	None	95/12,021	126/11,724	RR=0.74 (0.56 to 0.96)		⨁⨁⨁◯ Moderate	Critical
Logunov, 2021 ^19^ Vaccine: Gam-COVID- Vac rAd26-S/ rAd5-S	--	--	--	--	--	--	--	--	--	--		--	--

CI: Confidence interval; RR: Risk ratio Explanations

a. Data are from an interim analysis of the trial, with a short duration of follow-up. Estimates may change over a longer duration of follow-up.

b. The population included in the RCT may not represent all persons aged ≥16 years (BNT162b2) or ≥18 years (mRNA-1273; ChAdOx1; Gam-COVID-Vac rAd26-S/rAd6-S)

### Serious adverse event

**Table:** 

	Certainty assessment							No. of patients		Effect		Certainty		Importance
	No. of studies	Design	Risk of bias	I**nconsistency**	Indirectness	Imprecision	Other considerations	Vaccine	Placebo	Relative (95% CI)	Absolute (95% CI)			
Polack, 2020 ^15^ Vaccine: BNT162b2	1	Randomized trials	Not serious	Not serious	Serious^a b^	Very serious ^c^	None	126/21,621	111/21,631	RR=1.14 (0.88 to 1.46)		⨁◯◯◯ Very low		Critical
Baden, 2020 ^16^ Vaccine: mRNA-1273	1	Randomized trials	Not serious	Not serious	Serious^a b^	Very serious ^c^	None	147/15,185	153/15,166	RR=0.96 (0.77 to 1.20)		⨁◯◯◯ Very low		Critical
Voysey, 2020 ^17^^,^^18^ Vaccine: ChAdOx1	1	Randomized trials	Not serious	Not serious	Serious^a b^	Very serious ^c^	None	108/12,284	127/11,962	RR=0.83 (0. 64 to 1.07)		⨁◯◯◯ Very low		Critical
Logunov, 2021 ^19^ Vaccine: Gam-COVID- Vac rAd26-S/ rAd5-S	1	Randomized trials	Not serious	Not serious	Serious^a b^	Very serious ^c^	None	45/16,427	23/5,435	RR=0.65 (0. 39 to 1.07)		⨁◯◯◯ Very low		Critical

CI: Confidence interval; RR: Risk ratio Explanations

a. Data are from an interim analysis of the trial, with a short duration of follow-up. Estimates may change over a longer duration of follow-up.

b. The population included in the RCT may not represent all persons aged ≥16 years (BNT162b2) or ≥18 years (mRNA-1273; ChAdOx1; Gam-COVID-Vac rAd26-S/rAd5-S)

c. Imprecision was downgraded by 2 levels because the 95% of the relative risk (RR) was sufficiently wide that the estimate could include appreciable harm or benefit of the intervention. This outcome may be imprecise due to the small number of events reported during the observation period.

### All-cause mortality

**Table:** 

	Certainty assessment							No. of patients		Effect		Certainty	Importance
	No. of studies	Design	Risk of bias	I**nconsistency**	Indirectness	Imprecision	Other considerations	Vaccine	Placebo	Relative (95% CI)	Absolute (95% CI)		
Polack, 2020 ^15^ Vaccine: BNT162b2	1	Randomized trials	Not serious	Not serious	Serious^a b^	Very serious ^c^	None	2/21,621	4/21,631	RR=0.50 (0.09 to 2.73)		⨁◯◯◯ Very low	Critical
Baden, 2020 ^16^ Vaccine: mRNA-1273	1	Randomized trials	Not serious	Not serious	Serious^a b^	Very serious ^c^	None	6/15,185	7/15,166	RR=0.86 (0.29 to 2.55)		⨁◯◯◯ Very low	Critical
Voysey, 2020 ^17^^,^^18^ Vaccine: ChAdOx1	1	Randomized trials	Not serious	Not serious	Serious^a b^	Very serious ^c^	None	1/12,282	4/11,962	RR=0.49 (0.09 to 2.66)		⨁◯◯◯ Very low	Critical
Logunov, 2021 ^19^ Vaccine: Gam-COVID- Vac rAd26-S/ rAd5-S	1	Randomized trials	Not serious	Not serious	Serious^a b^	Very serious ^c^	None	3/16,427	1/5,435	RR=0.99 (0.10 to 9.54)		⨁◯◯◯ Very low	Critical

CI: Confidence interval; RR: Risk ratio Explanations

a. Data are from an interim analysis of the trial, with a short duration of follow-up. Estimates may change over a longer duration of follow-up.

b. The population included in the RCT may not represent all persons aged ≥16 years (BNT162b2); ≥18 years (mRNA-1273; ChAdOx1; Gam-COVID-Vac rAd26-S/rAd5-S)Imprecision was downgraded by 2 levels because the 95% of the relative risk (RR) was sufficiently wide that the estimate could include appreciable harm or benefit of the intervention. This outcome may be imprecise due to the small number of events reported during the observation period. CI: Confidence interval; RR: Risk ratio
